# Use of Relay Method for Enhancing Comb Construction by *Apis cerana cerana* Utilizing *Apis mellifera ligustica*

**DOI:** 10.3390/insects16010052

**Published:** 2025-01-08

**Authors:** Shunhua Yang, Shanglun Ge, Yiqiu Liu, Danyin Zhou, Xueyang Gong, Kun Dong

**Affiliations:** Yunnan Provincial Engineering and Research Center for Sustainable Utilization of Honeybee Resources, Eastern Bee Research Institute, College of Animal Science and Technology, Yunnan Agricultural University, Kunming 650201, China; fengxue_20141011@163.com (S.Y.); liuyiqiu2024@163.com (Y.L.); zhoudanyin1993@163.com (D.Z.)

**Keywords:** *Apis cerana cerana*, *Apis mellifera ligustica*, relay construct of comb, comb cell

## Abstract

Italian honey bees (IHBs, *Apis mellifera ligustica*) exhibit superior comb-building abilities compared with Chinese honey bees (CHBs, *Apis cerana cerana*), which often fail to fully utilize wax foundations, resulting in incomplete comb structures. The present study aimed to accelerate comb construction in CHB colonies using IHBs. In the experiment, IHB colonies, each with approximately 42,000 adult workers, required over four hours to construct a semi-drawn comb on CHB wax foundations. These semi-drawn combs were then transferred to experimental CHB colonies, where they were left to complete the comb-building process for an additional 24 h (4 + 24 h relay). In contrast, control CHB colonies were allowed to build combs on fresh CHB wax foundations for 28 h. The results showed that the combs built by CHBs in 28 h, those built by IHBs in 4 h, and the 4 + 24 h relay combs all shared a foundation size of 41.7 cm × 19.7 cm, with average thicknesses of 10.40 mm, 5.60 mm, and 12.20 mm, respectively. The average percentage of cells built in the 4 + 24 h relay combs was significantly higher than that in the 28 h combs. Additionally, cells in the relay combs were significantly larger and deeper than those in the combs built solely by CHBs. Although these findings prove that utilizing IHB colonies to form a base structure can enhance the efficiency of comb construction in CHB colonies, further research is needed to confirm the effects of 4 + 24 h operation on brood rearing and worker bee size.

## Simple Summary

The Italian honey bee (IHB, *Apis mellifera ligustica*) possesses a stronger ability to build combs than the Chinese honey bee (CHB, *Apis cerana cerana*) does. Consistent with this fact, beekeepers have found that the foundation near the bottom bars and sidebars of movable frames is not utilized in comb construction by CHB colonies. Therefore, this study used IHB to enhance comb construction by CHBs. We provided CHB colonies with a CHB comb foundation that was partially finished (4 h) by the IHB colonies to build a complete structure (24 h). The combs constructed via this interspecific relay method were better than those constructed by the CHB colonies. The CHBs did not build regular hexagonal cells on the wax comb foundation but followed their own patters to form what looked like a hexagon. Moreover, the CHBs maintained the preset cell size of the wax comb during relay comb construction. However, Chinese honey bees in the control group did not maintain the preset cell size. In other words, the technique adopted by CHBs in relay construction was different, but it enhanced speed and efficiency. These findings suggest utilizing IHB colonies to effectively compensate for the shortcomings of CHB colonies in comb construction. Implementing the technology discovered in this study will be conducive to efficiently keeping Chinese honey bee colonies.

## 1. Introduction

There are currently nine bee species in the genus *Apis*, with the Eastern honey bee and Western honey bee being the two most common ones [1]. Eastern honey bees are mainly distributed across Asia [2,3], while Western honey bees inhabit all continents except Antarctica and the Arctic [4]. The Chinese honey bee (CHB) and the Italian honey bee (IHB) are subspecies of the Eastern and Western honey bees, respectively. These subspecies belong to different bee species and exhibit reproductive isolation, which prevents interbreeding between them [5]. CHBs and IHBs also show biological differences. For instance, the cell diameters of Chinese worker, drone, and queen bees are, on average, 11.6%, 12.0%, and 6.0% smaller, respectively, than those of their Italian counterparts. Furthermore, the body length of Chinese worker bees is 1.86 mm shorter (approximately 86% of the body length of Italian worker bees), and their proboscis length is 1.20 mm shorter, representing about 81% of the proboscis length of Italian worker bees [6]. Additionally, CHBs weakly resist wax moths compared with IHBs [7]. Furthermore, the average daily syrup consumption of Italian worker bees is 68% higher than that of Chinese worker bees [8].

CHBs and IHBs naturally build cells of different sizes, averaging 4.83 and 5.49 mm in diameter, respectively [9]; this size difference is species-dependent. These natural differences in cell sizes inform the design of wax comb foundations. In 1857, Johannes Mehring produced the first wax comb foundation, now widely used in beekeeping [10]. Foundations serve as a basis for bees to build combs, with worker bees using the wax they secrete in combination with the foundation to complete the comb. Commercial wax foundations for CHBs and IHBs are preset to diameters of 4.75 and 5.30 mm, respectively, making them non-interchangeable between the two species.

Colony comb-building rates depend on the species [11], weather [1], nectar source [12], colony strength [5,13], and other factors. Surveys indicate that CHB comb-building is seasonal: spring yields 14,616 cm^2^ of comb, summer 3132 cm^2^, and autumn 8352 cm^2^ [14]. A wax foundation on a Langstroth frame measures 41.5 cm × 19.5 cm, with a two-sided area of 1618.5 cm^2^. In spring, CHB colonies with fewer than 12,900 worker bees exhibit low comb-building rates, whereas colonies with 17,200 to 30,100 bees construct a full frame within 4–5 days. However, colonies exceeding 36,400 bees either refrain from comb-building or build minimally due to swarming fever (due to the onset of swarming behavior) [15]. Beekeepers have observed that CHBs often leave the unbuilt wax foundation near the bottom and sidebars of frames, resulting in an incomplete comb area [16], leaving up to 25% of the total possible area unbuilt [17].

In summary, CHB colonies face three main disadvantages in comb construction. First, seasonal differences affect the colonies’ ability to build combs. Spring is optimal for comb construction, while efficiency is low in other seasons. Second, weak colonies either cannot build combs or build them at an extremely low rate, and strong colonies, due to swarming fever, also show low comb-building efficiency. Third, comb construction is deficient on the movable frame with a comb foundation, resulting in incomplete comb areas that do not reach the full frame area. Therefore, novel methods are needed to address the low comb construction rate in CHB movable-frame beekeeping.

On the other hand, IHBs, widely reared globally, build combs at a much higher rate than CHBs. As a result, some beekeepers add Langstroth movable frames with CHB comb foundations to IHB colonies, attempting to utilize IHB colonies to support comb construction for CHBs. In the early construction phase, the comb cell diameter remains the same. However, as construction progresses, IHBs alter the shape and size of their cells, resulting in cells that are unsuitable for either CHBs or IHBs [18]. IHBs, whose cells are 0.6–0.7 mm larger than those of CHBs, likely find it difficult to complete cells that are compatible with CHBs. Beekeepers recommend inspecting comb construction every 4–5 h after adding frames with CHB comb foundations to IHB colonies. The frames are removed as soon as IHBs add beeswax to the foundation base until the cell walls reach 1 mm in height. These semi-drawn combs built by IHB colonies can either be stored for future use or added directly to CHB colonies for expansion. The CHB queen will soon lay eggs in these cells, forming a complete brood nest within 2–3 days [19].

Although the studies mentioned above show that IHB colonies can help CHBs build worker combs, they provided only qualitative conclusions, lacking quantitative data. As a result, the observations lack a persuasive and theoretical basis, preventing bee farmers from effectively applying these strategies in their beekeeping practices. Therefore, the use of IHB colonies to assist CHBs in comb-building requires scientific validation and theoretical support to assess its feasibility and practicality.

## 2. Materials and Methods

### 2.1. Establishing Experimental Bee Colonies to Build Combs

From April to June 2023, limited, unidentified nectar sources were sporadically available within a 5 km radius of Yunnan Agricultural University in Kunming, Yunnan Province, requiring supplemental feeding for the experimental honey bee colonies. During this period, ambient temperatures ranged from 16 to 30 °C, with clear skies and stable weather conditions throughout the day. Daylight duration varied between 9 and 10 h, with minimal temperature fluctuations. In total, 35 CHB (*Apis cerana cerana*) colonies and 22 IHB (*Apis mellifera ligustica*) colonies were established as experimental colonies in Langstroth standard beehives, using normal egg-laying queens. The colony strengths among colonies of the same species were similar. Each CHB colony consisted of four combs with full adult worker bee coverage, while each IHB colony included a brood hive box containing ten frames and a super hive box with six combs, all similarly covered by adult worker bees. According to the method outlined by Guzman-Novoa et al. (2024) [20], the estimated density of adult worker bees per frame is approximately 4000 in CHB colonies and 2600 in IHB colonies. Therefore, the total population is estimated at approximately 16,000 adult worker bees for CHB colonies and 42,000 for IHB colonies. A pre-test was conducted with established IHB colonies before the formal experiment to assess comb construction on movable frames with CHB wax comb foundations. The pre-test procedure was as follows: at sunset, a Langstroth standard movable frame installed with a CHB comb foundation (average comb foundation size: 41.7 cm × 20.0 cm, average cell base count: 8624, cell base diameter: 4.75 ± 0.003 mm, the number cells, N_cell_ = 60) was added to the super hive box of an IHB colony for comb-building. Simultaneously, each colony was fed 1 kg of 50% sucrose syrup. From the addition of the frame with a foundation, comb construction by the IHB colony on the frame was checked every 2 h until the experimental frame was removed from the hive after 6 h, suspending further construction.

The thirty-five CHB colonies were divided into two groups, A and B. Group A (13 colonies) served as the control, and Group B (22 colonies) served as the treatment group. At sunset, a Langstroth standard movable frame with a CHB wax comb foundation was added to the hive of each CHB colony in Group A and the super hive box of each IHB colony as control and treatment frames, respectively, for comb-building. Each CHB colony in Group A and each IHB colony was fed 1 kg of 50% sucrose syrup. Four hours after adding the frame with the foundation, semi-drawn combs were promptly removed from the IHB hives and added to the hives of Group B colonies for CHB colonies to complete into finished combs. During this period, each CHB colony in Group B was fed 1 kg of 50% sucrose syrup, and 24 h after relay construction by the colonies, all experimental combs were removed from the hives of Group A and B colonies. As a result of the relay construction by CHB and IHB colonies, we obtained the following: after 28 h, combs were built by CHB colonies (colonies in Group A) on CHB foundations; after 4 h, semi-drawn combs were built by IHB colonies on CHB foundations; and after 4 + 24 h, finished combs were built by IHB and CHB colonies (colonies in Group B) in relay on CHB foundations.

### 2.2. Measurement of Comb Parameters and Cell Size

The ventral surface of the top bar on Langstroth standard movable frames is generally equipped with a structure to fix the comb foundation, which is a rectangular groove, measuring 4 mm wide and 3–4 mm deep, excavated longitudinally along the centerline of the top bar belly surface. When installing the comb foundation, the long edge of the foundation was embedded in the groove. During comb construction, worker bees added beeswax to the gaps in the groove to secure the comb foundation to the top bar of the movable frame. Therefore, the effective size of the comb foundation installed on the movable frame was 41.7 cm × 19.7 cm, with 8526 effective cell bases on both sides of the comb foundation installed in the movable frame. Actual comb lengths, widths, and thicknesses, as well as the number of cells on both sides of each comb, were recorded. The number of cells per comb is denoted by NC, and the number of effective cell bases (8526) on the comb foundation installed in the movable frame is denoted by ECB.

$$\mathrm{Cells}\;\mathrm{built}\;(\mathrm{percentage})=\frac{\mathrm{NC}}{\mathrm{ECB}}\times 100\%=\frac{\mathrm{NC}}{8526}\times 100\%$$

**Figure 1:**
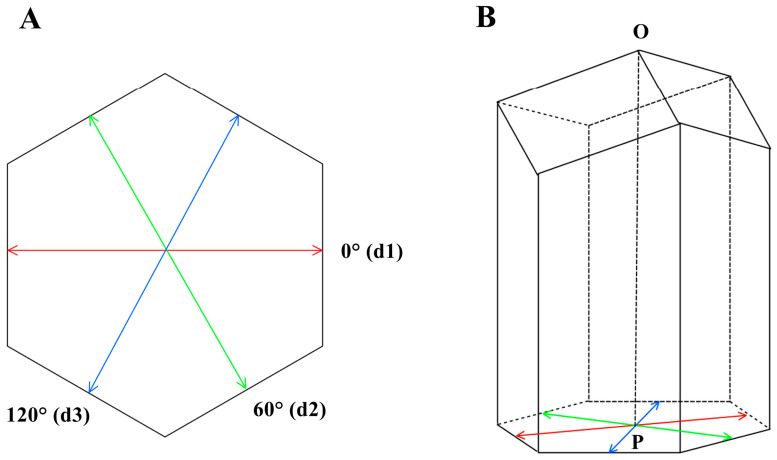
Cell diameters in different directions: (**A**) diameter d1 in the 0° direction, d2 in the 60° direction, and d3 in the 120° direction; (**B**) cell depth, OP: the straight-line distance from point O to point P.

Cell size was measured using epoxy resin cell molds according to Yang, Deng, Kuang, Zhou, Gong and Dong et al. [9]. Experimental combs were constructed by experimental bee colonies based on the CHB comb foundation. Three diameters of the cell mold and cell base were measured with a vernier caliper (accuracy ± 0.02 mm): diameter d1 in the 0° direction, diameter d2 in the 60° direction, and diameter d3 in the 120° direction (Figure 1A). The length of the mold represents cell depth (Figure 1B).

$$\mathrm{Average}\;\mathrm{cell}\;\mathrm{diameter}:\;d=\frac{d1+d2+d3}{3}$$

### 2.3. Statistical Analysis

Statistical analysis was performed using GraphPad Prism 9.5 (GraphPad Software, San Diego, CA, USA). Descriptive statistics were conducted on the actual comb length, width, thickness, and the number of cells built. The Shapiro–Wilk test was used to determine the normal distribution of experimental data for the percentage of cells built on the comb foundation and cell base, while the Kolmogorov–Smirnov test was applied to assess the normal distribution of cell size data. The one-way ANOVA test was applied to compare cell base diameters across different directions, with Tukey’s multiple comparisons used for diameters at 0° (d1), 60° (d2), and 120° (d3), while the Welch ANOVA test was employed to evaluate diameters after a 28 h build duration, with Games–Howell multiple comparisons applied to the same diameters (d1, d2, and d3). The Welch *t*-test was used to compare the average cell diameter across three directions and the depth of cells built by CHB colonies for 28 h, as well as the average cell diameter across three directions and cell depth in CHB and IHB colonies in relay for a duration of 4 + 24 h. The significance level was α = 0.05, and statistical values are expressed as mean ± standard error.

## 3. Results

### 3.1. Characteristics of Relay Comb Construction by CHB and IHB Colonies

**Figure 2:**
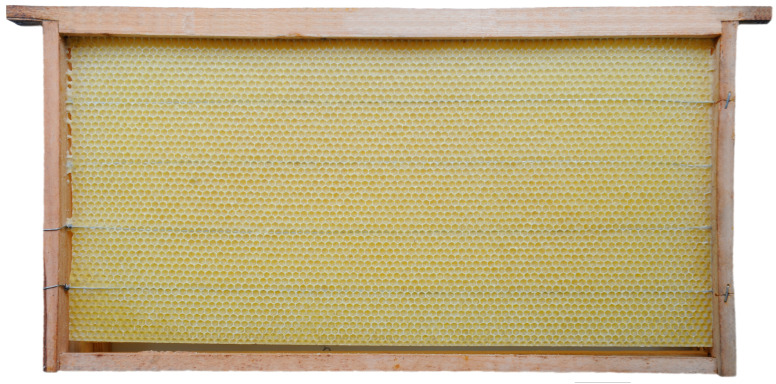
A semi-drawn comb constructed by an IHB colony using a movable frame with a Chinese worker bee comb foundation; the build time was four hours.

**Figure 3:**
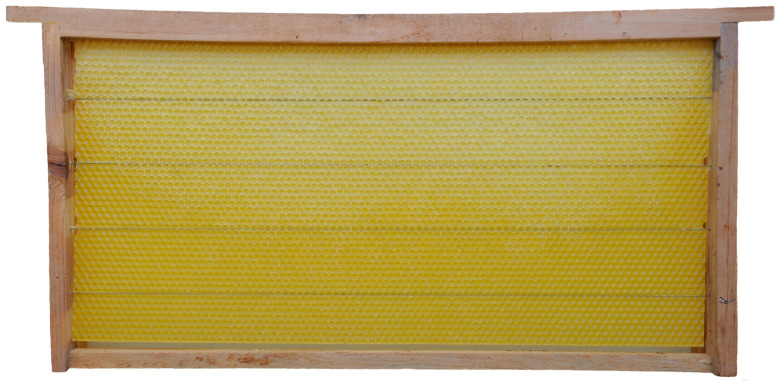
A semi-drawn comb constructed by an IHB colony using a movable frame with a Chinese worker bee comb foundation; the build time was less than four hours.

**Figure 4:**
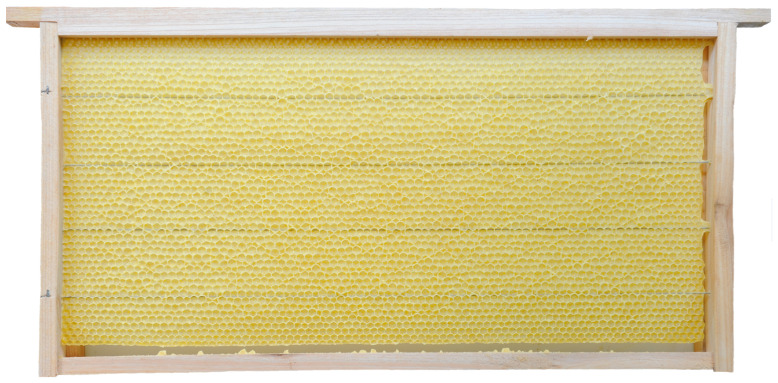
A semi-drawn comb constructed by an IHB colony using a movable frame with a Chinese worker bee comb foundation; the build time was more than four hours.

**Figure 5:**
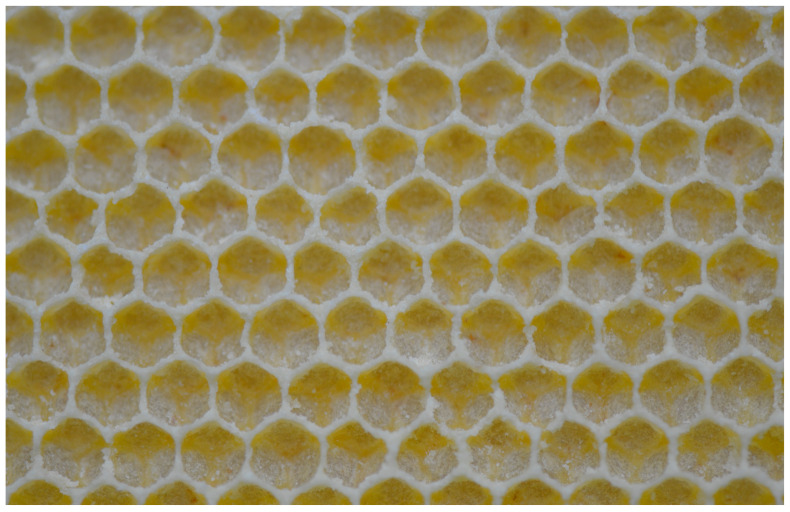
Beeswax particles on a semi-drawn comb.

**Figure 6:**
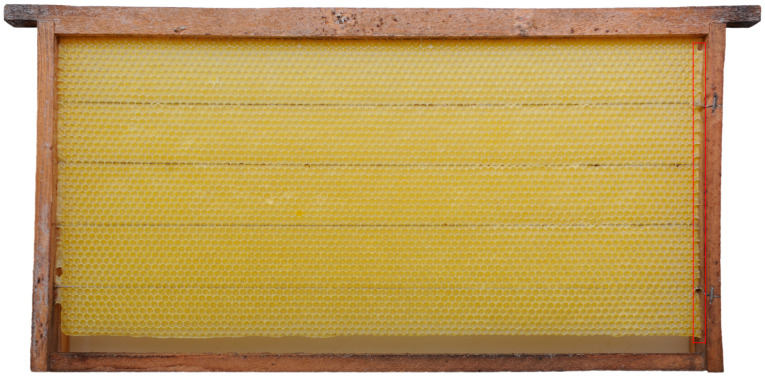
A 4 + 24 h comb constructed by an IHB colony in relay with a CHB colony. The red rectangular frame highlights the gaps that were filled by the bees.

**Figure 7:**
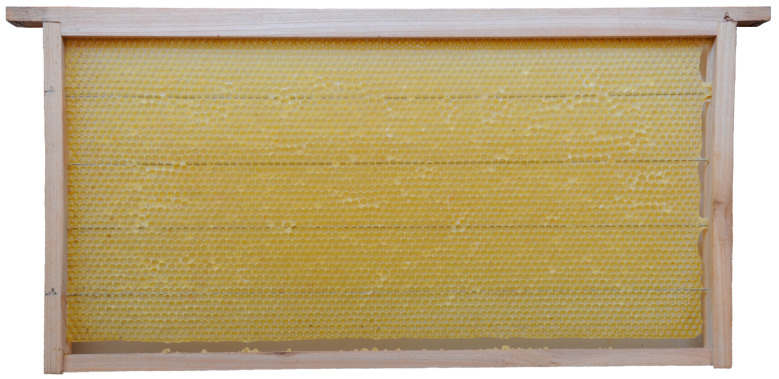
A finished comb constructed by an IHB colony in relay with a CHB colony; the construction time of the IHB colony was more than 4 h, while the construction time of the CHB colony was 24 h.

**Figure 8:**
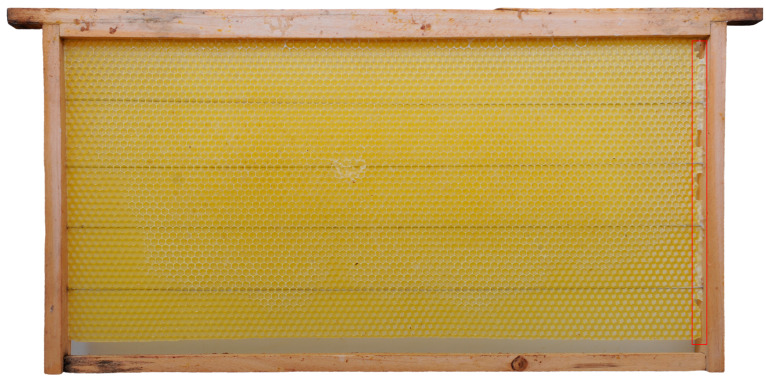
A 28 h comb constructed by a CHB colony using a movable frame with a Chinese worker bee comb foundation. The red rectangular frame highlights the gaps that were filled by the bees.

Honey bee colonies using movable frames with wax comb foundations typically begin building cells on both sides of the foundation simultaneously. The construction progresses from the top bar to the bottom bar of the movable frame, with colonies fed sugar syrup during construction. We found that the optimal time taken for an IHB colony with approximately 42,000 adult worker bees to build a semi-drawn comb using a movable frame with a CHB comb foundation was 4 h (Figure 2). When the construction time was less than 4 h, the number of cells on each side of the comb was reduced (Figure 3), while construction times exceeding 4 h altered the cell shape and size (Figure 4). Although IHB colonies required only 4 h to construct a semi-drawn comb with a CHB comb foundation, maintaining the intended cell shape and size, the surface of the comb was rough and had visible beeswax particles (Figure 5). However, after 24 h of relay building by CHB colonies, this semi-drawn comb with a rough surface transformed into a finished comb with a smooth surface (Figure 6) that was more appealing, on which the CHB queen began laying eggs. When we added semi-drawn combs with altered cell shapes and sizes to CHB colonies for 24 h, the comb was transformed into a finished comb with a smooth surface, but it lacked visual appeal (Figure 7). In this case, a large number of cells were restored to the preset shape and size, although a small portion could not be fully restored. Although the surfaces of combs built by CHB colonies after 28 h using CHB comb foundations were smooth and visually appealing, the cell bases on the left and right sides and bottom edges of each movable frame were incomplete (Figure 8).

### 3.2. Comb Size Parameters

**Figure 9:**
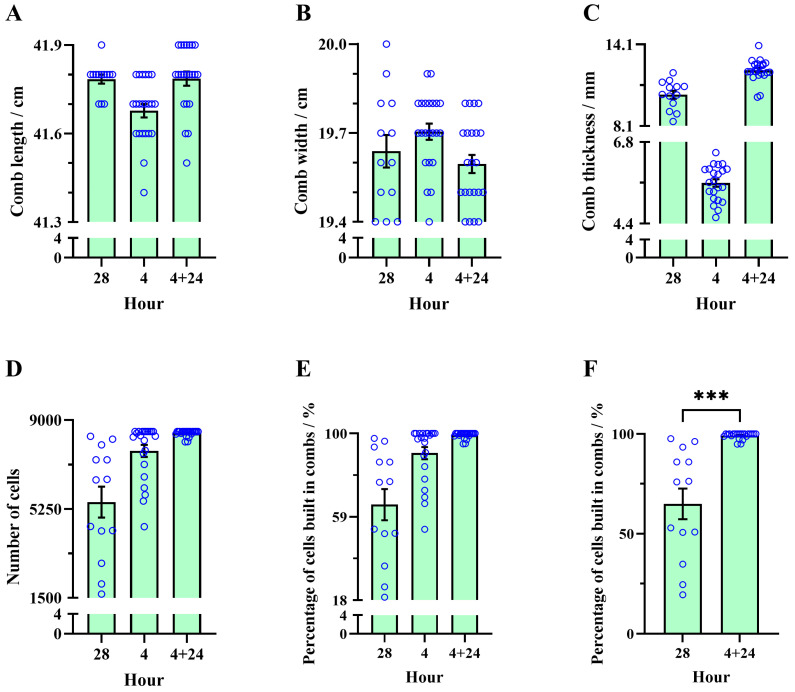
Comb parameters. (**A**) Comb length, (**B**) comb width, (**C**) comb thickness, (**D**) total number of cells on both sides of the comb, (**E**) percentage of cells built on the comb foundation, and (**F**) comparison of the percentage of cells built between 28 h combs and 4 + 24 h combs. The asterisk indicates significant differences (***, *p* < 0.001).

The average length, width, and thickness of combs built by CHB colonies over 28 h using CHB comb foundations were 41.78 ± 0.02 cm, 19.64 ± 0.05 cm, and 10.40 ± 0.29 mm, respectively. The data distribution shows that the percentage of constructed combs with a length greater than the average was 76.92%; the percentage of those with a width greater than the average was 46.15%; and the percentage of those with a thickness greater than the average was 61.54% (Figure 9A–C).

Likewise, the average length, width, and thickness of semi-drawn combs built by IHB colonies over 4 h using CHB comb foundations were 41.68 ± 0.02 cm, 19.70 ± 0.03 cm, and 5.60 ± 0.11 mm, respectively. The data distribution shows that the percentage of constructed combs with a length greater than the average was 63.64%; the percentage of those with a width greater than the average was 72.73%; and the percentage of those with a thickness greater than the average was 54.55% (Figure 9A–C).

The average length, width, and thickness of combs built by IHB colonies in relay with CHB colonies over 4 + 24 h using CHB comb foundations were 41.79 ± 0.02 cm, 19.60 ± 0.03 cm, and 12.20 ± 0.17 mm, respectively. The data distribution shows that the percentage of constructed combs with a length greater than the average was 72.73%; the percentage of those with a width greater than the average was 54.55%; and the percentage of those with a thickness greater than the average was 50% (Figure 9A–C).

### 3.3. Cell Construction Efficiency (Cells Built Percentage)

The average number of cells and percentage of cells built in combs by CHB colonies over 28 h using CHB worker comb foundations were 5540 ± 652.7 and 64.97 ± 7.66%, respectively. The data distribution shows that 53.85% of combs had a cell count above the average (5540 ± 652.7) or a cell build percentage of 64 ± 7.66% (Figure 9D,E).

The average number of cells and percentage of cells built in semi-drawn combs by IHB colonies over 4 h using CHB worker comb foundations were 7703 ± 255.3 and 90.35 ± 2.99%, respectively. The data distribution shows that 40.91% of semi-drawn combs reached 8526 cells or a 100% build percentage (based on 8526 effective cell bases installed in a movable frame) (Figure 9D,E).

The average number of cells and the percentage of cells built in combs by IHB colonies in relay with CHB colonies over 4 + 24 h using CHB worker comb foundations were 8451 ± 29.07 and 99.12 ± 0.34%, respectively. The data distribution indicates that 68.18% of combs had cell counts above the average (8451 ± 29.07) or a build percentage of 99.12 ± 0.34%; 63.64% of combs reached 8526 cells or a build percentage of 100% (Figure 9D,E).

The average percentage of cells built in combs (N_comb_ = 22) by IHB colonies in relay with CHB colonies over 4 + 24 h using CHB worker comb foundations was significantly higher than that in combs (N_comb_ = 13) by CHB colonies over 28 h using CHB worker comb foundations (Welch *t* test, t = 4.456, df = 12.05, *p* = 0.0008) (Figure 9F).

### 3.4. Comparison of Cell Sizes

**Figure 10:**
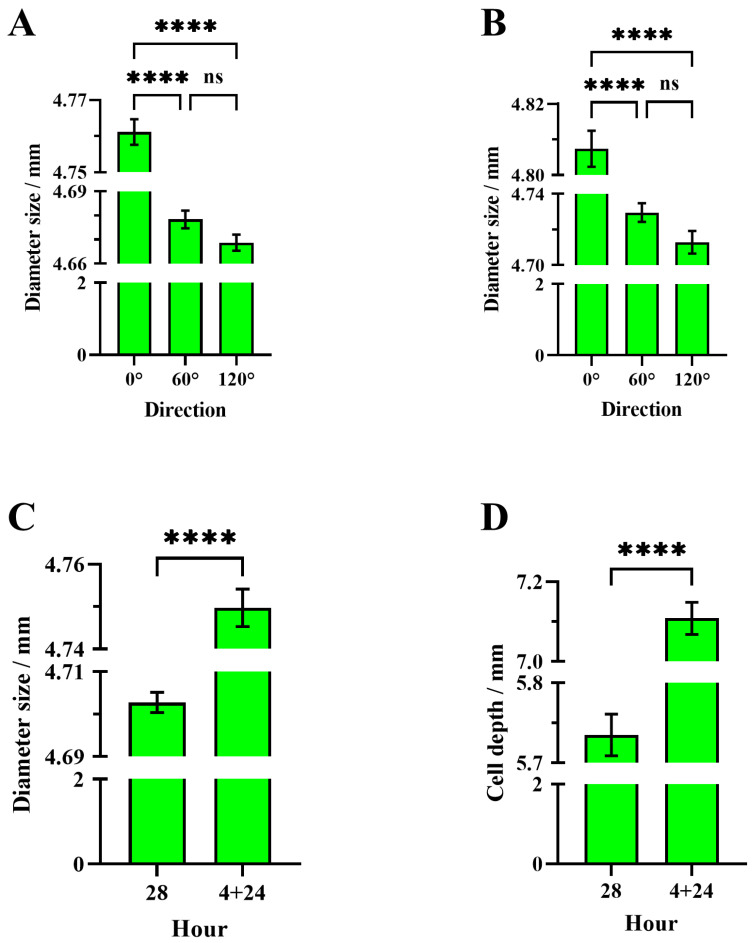
Cell parameters. (**A**) Comparison of diameters in different cell directions of a 28 h comb constructed by a CHB colony; (**B**) comparison of diameters in different cell directions of a 4 + 24 h comb constructed by an IHB colony in relay with a CHB colony; (**C**) comparison of the average cell diameter of a 28 h comb constructed by CHB colonies with the average cell diameter of 4 + 24 h comb constructed by IHB and CHB colonies in relay; (**D**) comparison of the cell depth of 28 h comb constructed by CHB colonies with that of 4 + 24 h relay combs constructed by IHB and CHB colonies. The asterisk indicates significant differences (****, *p* < 0.0001), while “ns” indicates not-significant differences (*p* > 0.05).

Significant differences were observed in cell diameters along three directions (0°, 60°, and 120°) with regard to the cells built by CHB colonies over 28 h using CHB worker comb foundations (one-way ANOVA, F = 210.6; df = 2, 1077; *p* < 0.0001). Tukey post hoc test results show that the average diameter d1 (4.76 ± 0.004 mm, N_cell_ = 360) in the 0° direction was significantly larger than d2 (4.68 ± 0.004 mm, N_cell_ = 360, *p* < 0.0001) in the 60° direction, and d3 (4.67 ± 0.003 mm, N_cell_ = 360, *p* < 0.0001) in the 120° direction. However, there was no significant difference (*p* = 0.1249) between d2 in the 60° direction and d3 in the 120° direction (Figure 10A).

Similarly, significant differences were found in cell diameters along three directions (0°, 60°, and 120°) with regard to cells built by IHB colonies in relay with CHB colonies over 4 + 24 h using CHB worker comb foundations (Welch’s ANOVA, W = 87.19; df = 2, 711.5; *p* < 0.0001). Games–Howell post hoc test results indicate that the average diameter d1 (4.81 ± 0.005 mm, N_cell_ = 360) in the 0° direction was significantly larger than d2 (4.73 ± 0.005 mm, N_cell_ = 360, *p* < 0.0001) in the 60° direction, and d3 (4.71 ± 0.006 mm, N_cell_ = 360, *p* < 0.0001) in the 120° direction. However, no significant difference (*p* = 0.1104) was observed between d2 in the 60° direction and d3 in the 120° direction (Figure 10B).

The average cell diameter (4.75 ± 0.004 mm, N_cell_ = 360) in cells built by IHB colonies in relay with CHB colonies over 4 + 24 h using CHB worker comb foundations was significantly larger than that of cells built by CHB colonies over 28 h using CHB worker comb foundations (4.70 ± 0.002 mm, N_cell_ = 360, Welch’s *t*-test, t = 9.372, df = 546.7, *p* < 0.0001) (Figure 10C).

The average cell depth (7.11 ± 0.040 mm, N_cell_ = 360) of cells built by IHB colonies in relay with CHB colonies over 4 + 24 h using CHB worker comb foundations was significantly greater than that of cells built by CHB colonies over 28 h using CHB worker comb foundations (5.74 ± 0.026 mm, N_cell_ = 360, Welch’s *t*-test, t = 28.57, df = 611.7, *p* < 0.0001) (Figure 10D).

One-way ANOVA test results show that there was no significant difference in the cell base diameters among the three directions (0°, 60°, and 120°) of the CHB comb foundation (F = 0.04; df = 2, 87; *p* = 0.96). The average diameters in the 0°, 60°, and 120° directions are 4.75 ± 0.0009 mm for d1, 4.75 ± 0.001 mm for d2, and 4.75 ± 0.001 mm for d3, respectively.

## 4. Discussion

### 4.1. Relay-Based Honeycomb Construction in IHB and CHB Colonies

The prerequisites for comb construction by IHB colonies in relay with CHB colonies include maintaining colony health and ensuring sufficient sugar syrup consumption during comb-building. To promote beeswax secretion and ensure the full participation of all worker bees in comb construction, three key conditions must be met within the hive [21,22]: first, a portion of the nest space must remain unoccupied by comb cells, allowing all worker bees capable of wax production to contribute; second, the right side of the hive, especially the area where young worker bees nurse broods, should remain free of the comb to stimulate wax scale production and optimize wax gland development. Third, the unoccupied space must not reduce the brood-rearing area or interfere with temperature and humidity, which are essential for maintaining brood health and nest integrity. Although our results show that an IHB colony with about 42,000 adult workers can build a movable frame with a CHB comb foundation into a semi-drawn comb within four hours, further investigation is needed to fully understand the dynamics. When an IHB colony exceeds 42,000 adult workers, increased worker participation further reduces the time required to complete semi-drawn combs on CHB comb foundations.

It is not entirely clear why IHB workers are able to build semi-drawn cells on CHB comb foundations that are smaller than their intended cell size. During cell construction, the antennae of IHB workers play a key role in measurement; the length of their antennae determines cell size and regularity [23]. The range of the antennae allows their tips to touch any point in front of the head [24]. With their forearms brought together, the antennae can also touch points on a smaller circle [25]. This ability enables bees to reach the opposite angles of the largest regular hexagons inscribed within these circles. Practically, bees construct two types of hexagonal cells: one for workers [26], with sides matching the forearm length of the antennae [1,27], and one for drones [5], with sides matching the full antenna length [1,27]. When IHB workers build cells on CHB comb foundations that are smaller than their set cell size, they may not use their antennae for measurement in the first four hours of construction. Thus, IHB colonies can create a semi-drawn CHB comb according to the preset cell size of CHBs. When the cell wall height reaches a certain threshold (5.60 ± 0.11 mm, ranging from 4.58 to 6.49 mm), IHB workers may then use their antennae to measure the cell wall length to correct construction errors, aligning with their standard cell size. Beyond four hours, the shape and size of cells in semi-drawn combs change.

Worker bees soften small beeswax particles by chewing and moistening them, then expanding these by adding more beeswax to form the bottom and walls of the cell [28]. Individual workers construct combs by removing excess materials and building cells where other workers have left off [29]. They inspect each other’s work and correct it when needed [30]. Special receptors on the antennae tips control cell wall thickness and smoothness. With antennae coordinating with mandibles, bees plane the cell walls. They control wall thickness by sensing dynamic, localized mandible movements, which are measured by antennae tips [31]. Honeybees follow the attachment–excavation theory for cell construction [32]. There are two worker types: attachers, who secrete and attach wax, and excavators, who remove it [33]. Worker bees may perform both roles, using antennae to locate attached beeswax, enabling them to excavate in darkness [34]. The cooperation between attachers and excavators leads to the rapid formation of honeybee comb cells [32,35].

On the one hand, the worker bees remove excess beeswax during cell construction to create a regular hexagonal shape, which partly determines the surface roughness of semi-drawn combs. Fresh beeswax particles found on the hive bottom board at the experimental comb location provide indirect evidence of this removal process. On the other hand, in natural comb-building without a comb foundation, the bottoms of cells are initially formed within 4 h after construction begins, and cell walls develop over 4 to 18 h. Using a four-dimensional X-ray microscope, researchers identified numerous micropores on cell walls and bases within 18 h [36], causing a rough surface on semi-drawn combs. The rough-surfaced semi-drawn combs built by IHB colonies on movable frames with CHB comb foundations within four hours resembled natural combs created by IHB colonies on movable frames without comb foundations over 18 h. When the construction time exceeded 18 h, micropores on natural comb cell surfaces diminished, and cell walls became smooth and straight due to cell compaction by builders over time [36]. This ultimately results in the transformation of the rough-surfaced comb into a delicate and attractive structure after 24 h of relay construction by the colonies.

Some researchers propose that the bee comb construction process can be explained by the stigmergy hypothesis, which suggests that comb-building behavior is guided by the presence of existing structures and previous actions [29,37,38,39]. In other words, each builder follows an algorithm that directs actions based on the developmental stage of the structure [29]. Builders transform the structure into a new form, which then prompts further actions by the builder or others until the structure is completed [30,39]. According to stigmergy theory, constructed cells guide workers to build subsequent cells [40]. This explains why CHB colonies can complete a delicate and beautiful comb on the rough, semi-drawn comb initiated by IHB colonies within four hours, using a movable frame with a CHB comb foundation. Stigmergy theory suggests that the rough-surfaced semi-drawn combs, constructed by IHB colonies on a movable frame with a CHB comb foundation, stimulate CHB builders to rapidly complete the comb, transforming it into a finished form.

### 4.2. Advantages of Relay Comb Construction Between CHBs and IHBs Colonies

The average lengths of the 28 h combs built by CHBs and the 4 + 24 h combs built by IHBs in relay with CHBs exceeded the lengths of the 4 h semi-drawn combs built by IHBs. This is because the comb foundation length (417 mm) is slightly smaller than the inner circumference of the movable frame (420 mm). However, after 28 h of construction, worker bees filled the 3 mm gap (shown in the red rectangle in Figure 6 and Figure 8). The average widths of the 28 h combs built by CHBs and the 4 + 24 h combs built by IHBs in relay with CHBs were smaller than those of the 4 h semi-drawn combs built by IHBs, likely due to the comb-gnawing biological trait of CHBs [41,42]. The lower edges of the 28- and 4 + 24 h combs showed slight nibbling marks (Figure 6 and Figure 8). The average thickness of the 4 + 24 h combs constructed in relay by IHBs and CHBs was greater than that of the 28 h combs built by CHBs, and the average cell count followed the pattern 4 + 24 h combs > 4 h combs > 28 h combs. Relay construction transformed more than 90% of the cell bases on the comb foundation into cells (Figure 9D), significantly increasing the cell coverage on comb foundations from 64.97% to 99.12% (Figure 9E). This indicates that relay construction between IHBs and CHBs provides a greater advantage than constructing combs alone, especially for CHB colonies.

### 4.3. Significance of Relay Comb Construction Between CHB and IHB Colonies

This study suggests that leveraging the strong comb-building capability of IHB colonies can effectively support CHB colonies. This helps in rapidly constructing combs for CHB queens to lay eggs. This approach addresses the weaker and slower comb-building ability of CHBs, enhancing the efficient maintenance of CHB colonies. Even with a comb foundation, we found that bees do not form perfect hexagons; the cell diameter in the 0° direction was significantly larger than that in the 60° and 120° directions, with no significant difference between the latter two (Figure 10A,B). This observation indicates that bees do not strictly follow the regular hexagonal template on the comb foundation. Instead, they create cells that resemble regular hexagons, a characteristic also seen in natural combs [9,43]. The ability to build visually regular hexagonal cells with high tolerance is beneficial for comb foundation manufacturing since bees can adapt to non-perfectly hexagonal shapes on the comb foundation.

The average cell diameter of the 4 + 24 h relay combs built by CHBs and IHBs was 4.75 mm, consistent with the cell base diameter on the comb foundation. This consistency indicates that both CHBs and IHBs maintained the preset cell size during relay comb construction. However, in 28 h combs built by CHB control groups, the average cell diameter was 4.70 mm (significantly smaller than 4.75 mm), suggesting that CHBs may not maintain preset cell sizes, possibly due to ecological factors. Prior studies indicate that the natural comb cell diameter varies by ecological environment and geographic location, with cell size increasing at higher altitudes [44,45]. The average cell depth of 4 + 24 h relay combs was 1.37 mm greater than that of 28 h CHB control combs, indicating that relay construction enhances CHBs’ cell wall heightening rate, improving their comb-building efficiency.

In beekeeping production practices, a professional apiary may manage over 100 colonies. Relay comb construction involves placing movable frames with wax comb foundations into the hives of IHB colonies for several hours before transferring them to the hives of CHB colonies. Although this method increases labor costs for beekeepers, it enables the rapid production of new combs in CHB colonies. By overcoming these initial labor costs, beekeepers can generate a large number of CHB new combs in a short period, which can then be sold as a commodity in the market.

## 5. Conclusions

An IHB colony with about 42,000 adult worker bees can build a movable frame with a CHB comb foundation into a semi-drawn comb in just four hours. The advantage of interspecific relay construction between IHB and CHB colonies is more efficient than independent comb construction within CHB colonies. CHBs did not construct regular hexagonal cells based on the template of the wax comb foundation. Instead, they built cells that visually resemble regular hexagons, following their own pattern. Both IHBs and CHBs maintained the preset cell size of the wax comb foundation during the 4 + 24 h relay comb construction period. However, CHBs in the control group did not maintain the preset cell size of the wax comb foundation. The relay construction technique effectively increases cell wall height at a faster rate and enhances comb construction efficiency.

## Data Availability

The preprocessed data that support the findings of this study are available from the corresponding author upon reasonable request.
